# Clinical value and survival analysis of subcutaneous soft tissue metastasis detected by ^18^F-FDG PET/CT

**DOI:** 10.3389/fonc.2025.1561137

**Published:** 2025-04-02

**Authors:** Yufei Gao, Hui Zhang, Tiancheng Hao, Lizhuo Jia, Jiangmeng Wu, Dongxue Wu, Siqi Wu, Yong Wang

**Affiliations:** ^1^ Department of Radiology and Nuclear Medicine, The First Hospital of Hebei Medical University, Shijiazhuang, China; ^2^ Department of Medical Imaging, Hebei General Hospital, Shijiazhuang, Hebei, China

**Keywords:** transfer, subcutaneous soft tissue, PET/CT, survival analysis, malignant tumor

## Abstract

**Objective:**

To retrospectively analyze cases of whole-body ^18^F-FDG PET/CT imaging, identify abnormal images of subcutaneous nodules, and explore the clinical application value of PET/CT in detecting subcutaneous soft tissue metastases and its impact on survival.

**Method:**

An analysis was conducted on cases from August 2019 to August 2024, and 57 cases of subcutaneous nodules were found to have positive 18F-FDG imaging, all of which underwent pathological puncture or biopsy. Collect patient histological subtypes, metastasis patterns, treatment, and survival rates. Kaplan Meier curves were used to estimate survival time, and Mantel-Cox univariate analysis was used to determine the correlation between extensive metastasis, degree of soft tissue involvement, systemic and local treatment, and survival time.

**Result:**

Among the 57 patients confirmed by pathology, 10 were benign and 47 were malignant. Among 47 cases of malignant tumors, 39 cases were postoperative cases, 8 cases were preoperative cases, and 4 cases had unknown primary lesions. A total of 88 subcutaneous lesions were found, including 28 patients with a single lesion, 12 patients with two lesions, and 8 patients with three or more lesions. One patient with unknown primary lesion reported abdominal wall nodules as the main symptom. PET/CT assisted in qualitative localization in 11 cases. Univariate analysis showed that the median survival time (22 months) of patients who did not receive local radiotherapy was significantly shorter than that of patients who did receive local radiotherapy (60 months) (P<0.05). There is no correlation between gender, metastasis pattern, metastasis at initial diagnosis, use of chemotherapy, and survival time.

**Conclusion:**

Whole body PET/CT imaging has important application value in the diagnosis of subcutaneous soft tissue metastasis. By comprehensively evaluating the patient’s overall condition and accurately determining whether there is a simple soft tissue metastasis, local intervention measures can bring more favorable prognosis for patients with good general condition and only soft tissue metastasis.

## 1 Introduction

Malignant tumors, as a type of invasive disease, are associated with local regional and distant spread. Prognosis depends greatly on local primary tumor invasion and the presence of distant metastases, which frequently occur in the liver, lungs, bones, adrenal glands, kidneys, and brain (1). Metastatic disease carries a poor prognosis of survival of approximately 5% at 5 years (2).

PET/CT technology has been developing since the late 1970s and has a history of over 40 years. This technology combines positron emission tomography (PET) and computed tomography (CT) to provide functional and structural information, which is of great value for the diagnosis and treatment evaluation of tumors. ^18^F-flurodeoxyglucose-PET/CT (^18^F-FDG-PET/CT) is considered an important tool for detecting and identifying tumors. It can help doctors detect the presence, size, and location of tumors, evaluate tumor staging, monitor tumor response to treatment, and detect recurrence (3). The scanning range of PET/CT is from the top of the skull to one-third of the thigh, which can cover the main organs of the human body. Engel et al. reported that the sensitivity of CT was 53.6%, while the sensitivity of PET was 82.5%. This indicates that PET/CT has high sensitivity in detecting tumors and metastases. For clinical and radiologists with metastatic lesions, their main focus is on important organs such as lymph nodes, bones, adrenal glands, and liver. Occasionally, abnormal FDG uptake may be found in subcutaneous lesions, and some patients may even have subcutaneous lesions as the initial symptom. PET/CT further discovers the primary lesion, so PET/CT is of great significance for finding the primary lesion, assessing treatment efficacy and prognosis through puncture localization (3–5). For all types of malignant tumors with soft tissue metastasis, treatment typically involves multidisciplinary collaboration, including chemotherapy for extensive metastasis and local treatment for symptomatic or well prognosis patients. With the advancement of tumor treatment, the prognosis of cancer patients has improved, which may change the metastasis pattern of the disease (6, 7). Nguyen et al. reported the effectiveness of PET/CT in detecting subcutaneous soft tissue metastases. 18F-FDG PET/CT can detect soft tissue metastases, including those that are not easily detected in traditional imaging techniques, but no further survival analysis has been conducted. So the researcher reviewed and analyzed cases from the past 5 years, intending to use PET/CT to preliminarily explore the abnormal uptake of subcutaneous FDG nodules and determine the factors that affect the treatment and survival of patients with primary malignant tumor soft tissue metastasis.

## 2 Data and methods

### 2.1 General information

A retrospective analysis was conducted on a substantial cohort of cases, encompassing a period from August 2019 through August 2024. The study population was carefully curated based on stringent inclusion and exclusion criteria to ensure the relevance and accuracy of the findings.

Inclusion Criteria: Patients were included in the analysis if they had undergone pathological puncture or biopsy, which confirmed the presence of a lesion. Additionally, the cases selected for this study exhibited clear image quality, as assessed by radiological standards. This criterion was critical to ensure that the PET/CT scans provided sufficient detail for accurate interpretation and subsequent analysis.

Exclusion Criteria: The study excluded patients with diabetes whose glucose levels exceeded 11 mmol/L. This threshold was chosen to minimize the impact of hyperglycemia on the PET/CT imaging results, as elevated glucose levels can affect the uptake of 18F-FDG, potentially leading to false-positive or false-negative findings. Furthermore, cases where urine contamination was clearly identified in the imaging were also excluded. Urine contamination can obscure the visibility of lesions and introduce artifacts that may compromise the diagnostic accuracy of the PET/CT scans.

57 confirmed cases were identified, with a total of 88 FDG abnormal uptake lesions. The researcher also extracted data on patient age, gender, abdominal wall nodule histology, primary and metastatic diagnosis dates, metastasis patterns, treatment methods, and death/last follow-up dates. This study has been ethically approved by the Institutional Ethics Committee (Ethics Committee of the First Hospital of Hebei Medical University, June 32, 2020) and complies with local regulations in China.

### 2.2 Image acquisition

All patients should fast for at least 6 hours before undergoing PET/CT examination. After ensuring a normal blood glucose level, the patient received intravenous injection of 0.10-0.15mCi/kg (3.7-5.5MBq/kg) of 18F-FDG, followed by resting for 50-60 minutes and image acquisition. Use an integrated PET/CT (Biographm CT · S, Siemens, Germany) for imaging. Low dose CT is used to cover one-third of the area from the head to the thighs, and PET data is obtained in three-dimensional mode. Each bed lasts for 2 minutes and has 6-8 beds. Some patients have delayed local imaging of the lesion 2 hours after the completion of image acquisition.

### 2.3 Image analysis

Firstly, a positive lesion was evaluated through visual imaging, which presented as a subcutaneous localized FDG uptake increase lesion. CT images showed nodular soft tissue density shadows. Manually delineate the region of interest for subcutaneous positive lesions on PET/CT images to obtain SUVmax values. SUVmax values were generated for all lesions included in this study. The SUVmax (maximum standard uptake value) of the lesion>2.5, the CT scan on the same machine showed a positive result for local density changes.

### 2.4 Statistical methods

SPSS 26.0 software was used for statistical analysis of the data. Kaplan Meier curves were used for survival analysis to describe the survival time after diagnosis of malignant tumors and soft tissue metastases. Mantel Cox univariate analysis was used to determine the correlation between extensive metastasis, degree of soft tissue involvement, systemic and local treatment, and survival time. P<0.05 indicates statistical significance of the difference.

## 3 Results

There were 57 patients confirmed by pathology, with an average age of (57.7 ± 11.7) years, including 36 males and 21 females. Among them, there were 10 benign cases and 47 malignant cases. 10 benign cases, 4 cases of subcutaneous granuloma, 4 cases of surgical incision inflammation or granulation tissue, and 2 cases of lipoma or other benign lesions. Benign cases are more common in granulomas, presenting as single or multiple nodular FDG uptake lesions with a maximum SUVmax value of 3.8.

Among 47 patients with malignant tumors, 39 cases were treated after tumor resection, including 10 cases of primary colon cancer, 9 cases of gastric cancer, 3 cases of rectal cancer, 2 cases of ovarian cancer, 2 cases of liver cancer, 1 case of renal cancer, 2 cases of appendix cancer, 1 case of small intestine cancer, 5 cases of lung cancer, 1 case of breast cancer, 1 case of bladder cancer, 1 case of prostate cancer, and 1 case of femur cancer. The primary tumor has been confirmed, with 8 non-surgical cases, including 2 cases of lung cancer, 1 case of malignant melanoma, 1 case of neurofibromatosis, 1 case of malignant schwannoma, 2 cases of lymphoma, and 1 case of mesothelioma. 28 patients had a single subcutaneous nodule, 11 had two nodules, and 9 had three or more nodules. Fifteen lesions were located near the surgical incision or stoma, mainly in cases of colorectal cancer; 5 cases were located in the puncture drainage pathway; Four patients with unknown primary lesions reported subcutaneous nodules as the main symptom, and PET/CT assisted in qualitative localization in 18 cases. The diagnostic staging of malignant tumors is: stage II in 12 cases; 31 cases in Phase III; Four cases in stage IV. Soft tissue metastasis occurs after diagnosis of malignant tumors, with a median age of 59 years (range 27-84). After diagnosis of primary malignant tumor, soft tissue metastasis occurred at a median of 6.0 months (range 3-9) (**Tables 1** and **2**).

**Table 1:** Characteristics of 47 patients with abdominal soft tissue metastasis.

Characteristics	N (%)
Age at soft-tissue metastasis diagnosis (years)	59 (27-84)^a^
Time to soft-tissue metastasis diagnosis after primary cancer diagnosis (months)	6 (3-10)^a^
Female	30 (63.8)
Male	17 (36.2)
Primary histological subtype	
Squamous cell carcinoma	4 (8.5)
Adenocarcinoma	33 (70.2)
Neuroendocrine carcinoma	1 (2.1)
Clear cell carcinoma	1 (2.1)
Transitional cell carcinoma	1 (2.1)
Melanoma	1 (2.1)
Mesothelioma	1 (2.1)
Malignant schwannoma	1 (2.1)
Lymphoma	2 (4.3)
Malignant neurofibromatosis	1 (2.1)
Osteosarcoma	1 (2.1)
Cancer stage at diagnosis	
II	12 (25.5)
III	31 (65.9)
IV	4 (8.5)

a Data presented as median (range).

**Table 2:** Clinical characteristics of 47 cases of soft tissue metastases.

Feature	N (%)
Scope of lesion metastasis	
Widespread systemic metastasis	41 (87.2)
Only abdominal wall soft tissue metastasis	6 (12.8)
Symptomatic soft tissue lesions	
Yes	10 (21.3)
No	37 (78.7)
Number of soft tissue nodule metastases	
Solitary	28 (59.6)
Multiple	19 (40.4)

**Figure 1:**
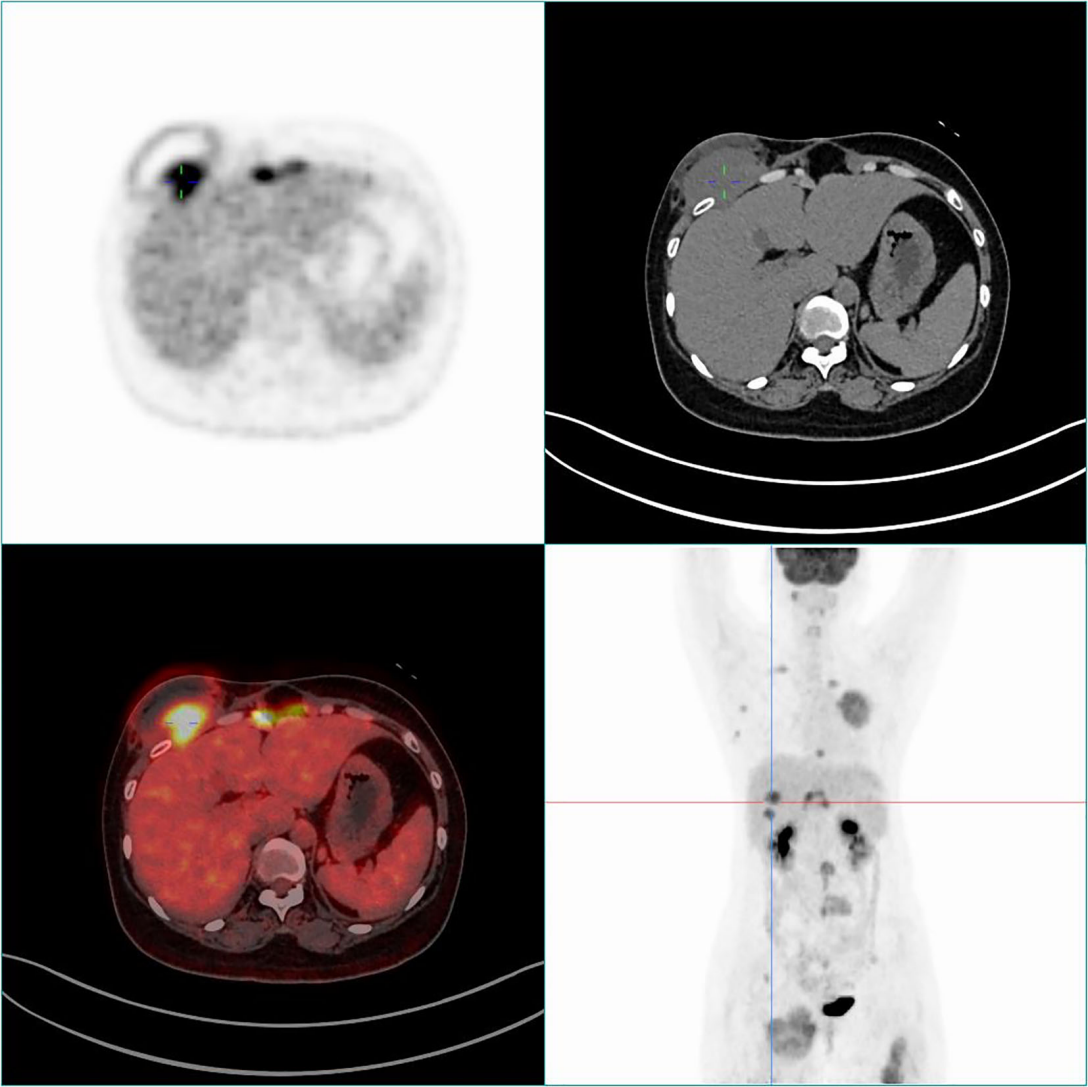
PET/CT scan of subcutaneous soft tissue metastasis in the right anterior chest of a 40 year old female patient. The whole-body maximum intensity projection (MIP) and fusion PET/CT and CT axial images showed an FDG positive nodule in the right anterior chest (SUVmax=8.8). Pathological indication: malignant schwannoma.

**Figure 2:**
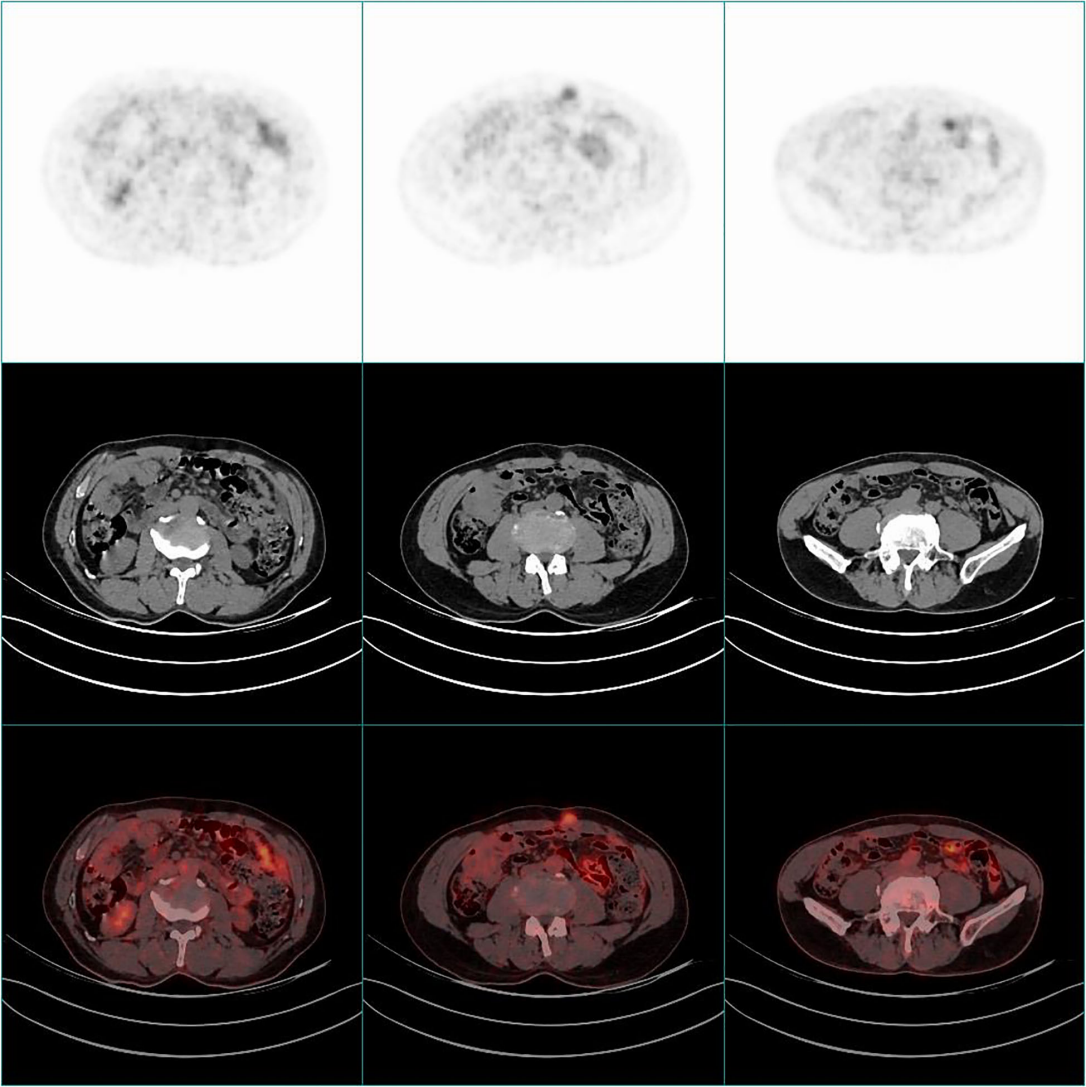
PET/CT scan of a 69 year old male patient with soft tissue metastasis in the anterior abdominal wall. PET images and fusion PET/CT and CT axial images showed a FDG positive nodule (SUVmax=3.4) near the anterior abdominal wall navel, accompanied by diffuse thickening of the peritoneum and increased FDG uptake. Pathological indication: malignant mesothelioma.

77 FDG positive subcutaneous metastases with an average SUVmax value of (8.92 ± 5.85). The largest lesion volume was in a patient with an unknown cause of abdominal wall mass, with a cross-sectional diameter of approximately 7.0 × 4.6cm and a SUVmax of approximately 8.7. Pathological puncture results showed malignant schwannoma (**Figure 1**). Another case was an unexpected discovery of an abdominal wall nodule with a maximum SUV value of 3.4 and a diameter of approximately 2.2cm. Pathological puncture results showed that it originated from malignant mesothelioma, and whole-body PET/CT showed diffuse thickening of the peritoneum with high FDG uptake (**Figure 2**).

**Figure 3:**
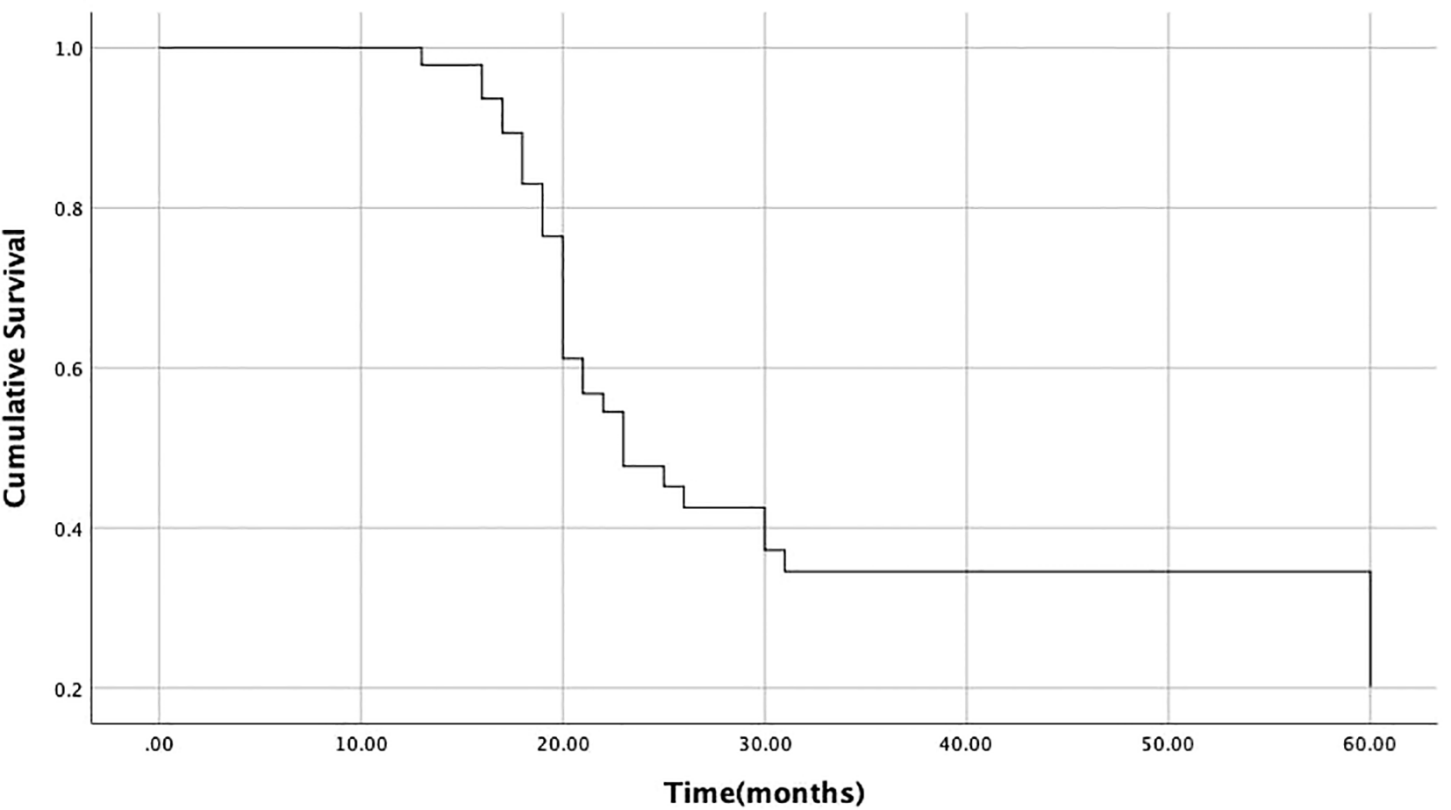
Survival time of 47 patients after diagnosis of soft tissue metastasis (median survival time is 23 months).

**Figure 4:**
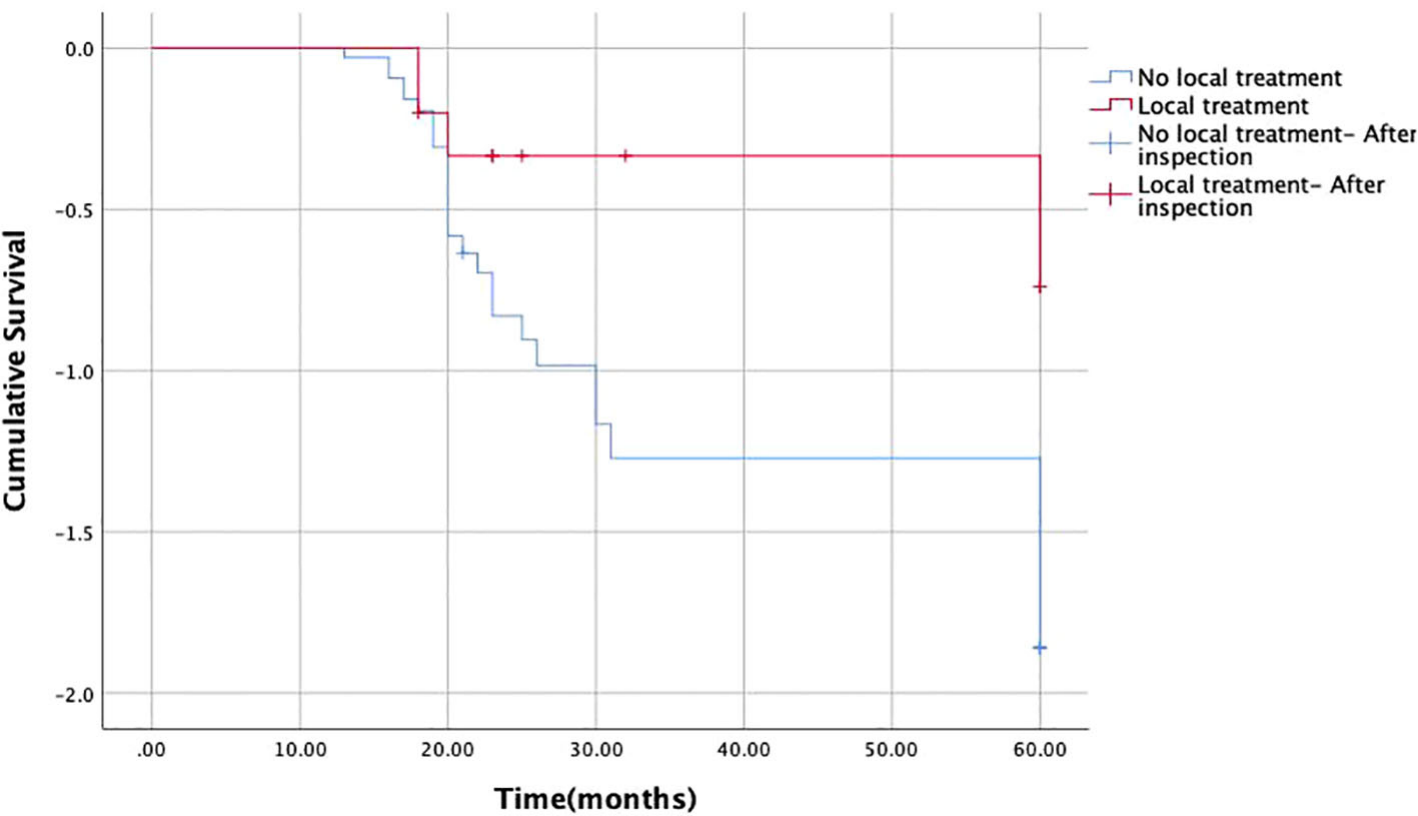
Survival time after diagnosis of soft tissue metastasis: local radiotherapy and no local radiotherapy.

47 patients have treatment information. Systemic chemotherapy was the main treatment method (n=34) and the only treatment intervention among the 29 patients. Among the patients who did not receive systemic chemotherapy, 5 patients received both systemic chemotherapy and local treatment. 11 patients received local treatment for metastatic lesions without systemic treatment. The most common local interventions are radiotherapy alone (n=5) or radiotherapy combined with surgical resection (n=3), followed by surgical resection alone (n=3) (**Table 3**). During follow-up, among the 11 patients considered for local intervention, 4 reported improvement and 5 reported complete pain relief. The remaining 2 patients reported no improvement in pain. Among patients diagnosed with stage IV malignant tumors, one patient (25%) received local intervention due to soft tissue metastasis. Two older patients did not receive intervention treatment. The median survival time after diagnosis of soft tissue metastasis was 23.0 months (**Figure 3**). Univariate analysis showed that the median survival time (22 months) of patients who did not receive local radiotherapy was significantly shorter than that of patients who did receive local radiotherapy (60 months) (P<0.05) (**Table 4**, **Figure 4**). There is no correlation between gender, metastasis pattern, metastasis at initial diagnosis, use of chemotherapy, and survival time.

**Table 3:** Treatment types of 47 patients with abdominal soft tissue metastasis.

Treatment type	N (%)
Chemotherapy	34 (72.3)
Chemotherapy only	29 (61.7)
Local therapy	
Radiation only	5 (10.6)
Surgical resection only	4 (8.5)
Radiation and surgical resection	2 (4.3)
None	2 (4.3)

**Table 4:** Survival time after diagnosis of 47 cases of soft tissue metastasis.

Parameter	Survival (months)		*P* value
	Median (SE)	95% CI	
Overall	23.0 (3.0)	17.1-28.9	–
Sex			0.928
Male	25.0 (3.0)	19.1-30.9	
Female	22.0 (2.7)	16.8-27.2	
Metastatic pattern			0.132
Soft-tissue metastases only	60.0 (12.4)	35.7-84.3	
Widespread metastases	23.0 (1.5)	20.0-25.9	
Metastases at initial cancer diagnosis			0.594
Yes	22.0 (3.2)	15.8-28.2	
No	23.0 (3.0)	17.1-28.9	
Number of soft-tissue lesions			0.001
Single	30 (3.8)	22.6-37.4	
Multiple	20 (1.6)	16.8-23.2	
Radiation	60.0 (30.4)	(0.4-119.6)	0.034
Chemotherapy	22.0 (2.1)	(17.9-26.1)	0.605
Surgery	–	–	0.827

## Discussion

Soft tissue metastasis of tumors is relatively rare, mainly occurring in muscles and subcutaneous soft tissues. Subcutaneous soft tissue metastasis is even rarer, more common in adenocarcinoma metastasis (6–8), which is consistent with our research and may be related to the fact that the primary tumors that undergo soft tissue metastasis mostly come from digestive tract tumors. Xiao et al. (4, 5, 8–12) reported that most patients have subcutaneous soft tissue metastases located on the chest or abdominal wall. Plaza et al. (13) also confirmed this conclusion and pointed out that the first four soft tissue metastases were abdominal wall, back, thigh, and chest wall. Wang et al. (14)have reported that cases of soft tissue metastasis suggest that the tumor may be in the late or advanced stage and can serve as an independent factor affecting prognosis. Although the specific mechanism of soft tissue metastasis has not been fully elucidated, from a macroscopic perspective, it may be related to lymph node or hematogenous metastasis, as well as implant metastasis through endoscopic surgical approach; From a microscopic perspective, it may be related to the pH value, temperature, and accumulation of metabolites in local soft tissue (15–17). Clarifying these mechanisms can help prevent distant metastasis of tumors and provide new strategies for clinical treatment, thereby improving prognosis. In the current limited understanding of the mechanism of soft tissue metastasis, early detection of soft tissue metastasis is of great significance for judging tumor recurrence, systemic and lymph node metastasis, helping to clarify staging and guide treatment, and significantly improving patient prognosis.

PET/CT, as a whole-body functional metabolic imaging, is not only used for tumor staging, re staging, and post treatment efficacy observation, but also for detecting systemic metastases, especially those hidden lesions without morphological changes in traditional imaging (3). Through our retrospective analysis, the researcher found that there were many patients whose tumor markers continued to increase in laboratory tests, but traditional imaging did not find lesions. The 77 subcutaneous FDG high uptake nodules discovered in this study were derived from different tumors, some of which underwent minimally invasive surgery through the abdominal wall and may have metastasized during the surgical path due to improper operation. This study found 20 cases of implant metastasis at the surgical incision and fistula site. This study unexpectedly discovered several cases of abdominal wall nodule metastasis in patients without pain symptoms and without palpable masses. Due to the good results of PET/CT in tumor staging and post-treatment evaluation in recent years, this may be one factor explaining the increase in reports of asymptomatic soft tissue metastasis.

The treatment plan for patients with soft tissue metastasis is diverse and needs to be developed based on the specific situation and prognosis of the patient. The treatment guidelines for soft tissue metastasis recommend chemotherapy as the primary treatment for patients with generally acceptable conditions and extensive metastasis. Because soft tissue metastasis usually does not cause functional damage or direct risk of death, the general consensus is that local intervention should be limited to symptomatic patients with good prognosis. Radiotherapy has been recommended for patients with symptomatic diseases with good prognosis, while surgical resection is only recommended for patients with symptomatic and local diseases with good prognosis (18, 19). The treatment data on soft tissue metastasis of malignant tumors in Pogkas et al. (6, 7, 20, 21) show that most patients received systemic chemotherapy, and some patients also received local treatment. In our series of studies, patients who received local treatment had a much longer survival time than those who did not receive local treatment. Therefore, local intervention is necessary for patients with good prognosis and symptomatic soft tissue diseases, and is associated with improving functional prognosis. Soft tissue metastasis is a hallmark of systemic diseases, and regardless of the primary malignant tumor, it is associated with poor prognosis. Pop et al. (22) showed that the median survival time after skeletal muscle metastasis was 6 months, which is much shorter than the median survival time for diagnosing subcutaneous soft tissue metastasis (23 months). From El et al. (8), the researcher found a study on survival analysis after diagnosis of soft tissue metastasis in esophageal cancer. The study showed that the median survival time after diagnosis of soft tissue metastasis in esophageal cancer was 8.9 months [(0.2-17.6) 95% CI], which is much shorter than our research results. This may be related to the increasing application of PET/CT and the improvement of clinical treatment plans. In our study, the median survival time after diagnosis of soft tissue metastasis was 23.0 months [(17.1-28.9) 95% CI]. These times are similar to earlier research reports describing survival after skeletal muscle metastasis in all types of cancer. Meanwhile, the researcher also found that patients with malignant tumors that only metastasize to soft tissues have significantly longer survival times than those with extensive metastasis. Compared with not undergoing local radiotherapy, the survival time of patients undergoing local radiotherapy is significantly prolonged. For patients with only soft tissue metastases, an active local treatment strategy may help them achieve long-term asymptomatic survival.

This study has certain limitations: 1 There are relatively few research samples and a large number of pathological types in the samples, and no targeted analysis has been conducted on the soft tissue metastasis of a pathological type of malignant tumor. Therefore, in the next step of our research, the researcher will analyze the significance of soft tissue metastasis in a pathological type of malignant tumor in a more detailed and specific manner. For survival analysis, a 5-year follow-up period may be a bit short and further follow-up is needed to achieve more accurate survival time. Despite these limitations, our study remains the most comprehensive assessment of this rare event. Our future research aims to use molecular imaging to help identify factors that induce soft tissue metastasis in patients with malignant tumors.

## 4 Conclusion

PET-CT, as a cutting-edge imaging technique, has demonstrated its unique value in diagnosing soft tissue metastases. It can comprehensively evaluate the patient’s overall condition and accurately determine whether there is a simple soft tissue metastasis. Based on this information, we can determine that for patients who are generally in good condition and only have soft tissue metastases, local intervention measures can bring more favorable prognosis outcomes.

## Data Availability

The original contributions presented in the study are included in the article/supplementary material. Further inquiries can be directed to the corresponding author.
